# Intracardiac thrombosis in Behçet's disease: a rare complication

**DOI:** 10.11604/pamj.2013.15.91.2635

**Published:** 2013-07-09

**Authors:** Ghita Saghi, Nawal Doghmi

**Affiliations:** 1 Cardiologie B, CHU IBN SINA, Rabat, Maroc

**Keywords:** Thrombosis, Behçet's disease, pulmonary embolism

## Images in medicine

A 13 year-old boy with BD since 2007 was admitted in May 2012 for dyspnea and prolonged fever for 2 months. A physical examination did not reveal signs of heart failure. Transthoracic echocardiography showed a homogeneous mass attached to the right ventricle. We complemented with a magnetic resonance Imaging scan (MRI) that revealed a voluminous, mobile thrombus in the right ventricle and a massive pulmonary embolism. We opted for a conservative treatment: Heparin, oral anticoagulation and Methylprednisolone followed by Prednisone. The outcome was favorable under medical treatment. Cardiovascular disease in Behçet's disease varies from 7 to 29% of reported cases and is represented mainly by endocarditis, pericarditis and myocardial infarction. Intracardiac thrombosis (ICT) is exceptional and can since the first case described at necropsy by Buge in 1977, only about 50 cases of ICT have been reported. The association with pulmonary embolism is serious and life-threatening.

**Figure 1:**
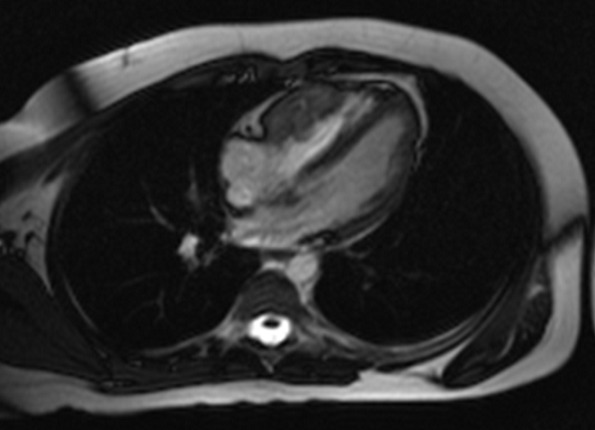
Cardiac magnetic resonance image, heart four chambers view showing right ventricle thrombus (arrow)

